# Optimal cutoff value of the Japanese shortened version of the Pediatric Symptom Checklist (PSC) youth self-report

**DOI:** 10.1186/s40359-026-04652-w

**Published:** 2026-04-28

**Authors:** Takahiro Higuchi, Yuko Ishizaki, Hiroyuki Uenishi, Yoshitoki Yanagimoto, Mayuko Ono, Haruhiko Ishida, Kazunari Kaneko

**Affiliations:** 1 Department of Pediatrics, Kansai Medical University, Shinmachi 2-3-1, Hirakata, Osaka 573-1010 Japan; 2 Graduate School of Psychology, Kansai University, Yamatecho 3-3-35, Suita, Osaka 564-8680 Japan; 3 Department of Psychology and Social Welfare, Osaka Ohtani University, Nishikiorikita 3-11-1, Tondabayashi, Osaka 584-8540 Japan; 4 Department of Pediatrics, Kansai Medical University Medical Center, Fumizono-cho 10-15, Moriguchi, Osaka 570-8507 Japan

**Keywords:** JPSC-17-Y, Screening, Schoolchildren, Psychosocial problems, Early intervention, Prevention

## Abstract

**Background:**

The Pediatric Symptom Checklist (PSC) is a brief questionnaire aimed at detecting psychosocial problems of children and adolescents for intervention in the early stage. Though we have devised a 17-item version of the PSC-Youth Self-Report (PSC-17-Y) Japanese version, our subsequent goal was to evaluate its adequacy by comparing the scores of patients and of schoolchildren for the prevention and early intervention of schoolchildren’s psychosocial problems.

**Methods:**

This study was conducted in Osaka, Japan’s second-largest city, between July 2020 and September 2022, during which participants completed the Japanese version of the PSC-17-Y (JPSC-17-Y). The participants were 52 patients with psychosocial problems (23 boys and 29 girls; mean age, 13.6 years) and 64 age-matched schoolchildren (24 boys and 40 girls; mean age, 13.4 years). The total score and each factor score of the JPSC-17-Y were compared, with the Mann-Whitney U test used to examine the differences between the groups.

**Results:**

The results showed that the patient group’s scores were significantly higher than the control group’s for all factors and the total score. To estimate the cutoff value for differentiating between the control group and the patient group using the total score of the JPSC-17-Y, the Area Under the Curve (AUC) of the Receiver Operating Characteristic (ROC) curve was calculated. The AUC was 0.755, indicating a moderate level of predictive performance for the JPSC-17-Y. The cutoff value of the total score between 12 and 13 was considered clinically appropriate.

**Conclusions:**

These findings suggest that the JPSC-17-Y can lead to prevention of and early intervention for psychosocial problems of schoolchildren.

**Supplementary Information:**

The online version contains supplementary material available at 10.1186/s40359-026-04652-w.

## Background

Despite the decrease in COVID-19 cases, the rate of youth suicides remains high in Japan, which among the Group of Seven (G7) nations, stands out as the only developed country where suicide is the leading cause of death of the younger generation, particularly teenagers and those in their 20s [1]. Moreover, the number of Japanese children unable to attend school has exceeded 290,000 and continues to increase [2]. Immediate assessment and care are necessary to prevent the onset of mental illness and suicide in affected children; however, the current system for assessing and providing care is inadequate. Although parents may be capable of accurately assessing their children’s behavioral problems, evaluating their mental health, including depression and anxiety, proves to be challenging [3]. Thus, it is essential to develop a straightforward self-report scale for children to assess their mental health, not only for the post-COVID-19 era, but also for potential future pandemics.

The Pediatric Symptom Checklist (PSC) [4–8] is a 35-item screening questionnaire that is completed by parents and designed to help pediatricians in outpatient practice identify school-age and preschool-age children [9] with difficulties in psychosocial functioning. The PSC covers a broad range of emotional and behavioral problems and is meant to provide an assessment of psychosocial functioning. The PSC and a 17-item version of the parent completed PSC form (PSC-17) [10–11] have been translated into more than 20 languages and have been widely used in both research and clinical settings. Since its development in 2000, the PSC-Youth Self-Report (PSC-Y) has been used in more than 20 studies, with most reporting positive screening rates ranging from 4.2% to 20.0% across diverse samples drawn from schools, outpatient pediatric practices, and other community-based settings [12–17]. In addition, the short form, 17-item version of the PSC-Youth Self-Report (PSC-17-Y) [18] is a validated measure that assesses psychosocial problems overall and in three major psychopathological domains (internalizing, externalizing, and attention-deficit/hyperactivity disorder), taking 5–10 min to complete. The PSC-17-Y has also been translated into several languages [19–21]. Ishizaki et al. [22] translated the PSC, and Hiratani et al. [23] translated the PSC-17 into Japanese. Moreover, Higuchi et al. [24] translated the PSC-17-Y into Japanese and preliminarily demonstrated that the Japanese version of the PSC-17-Y (JPSC-17-Y) was a reliable and valid screening tool that could be useful in detecting psychosocial problems in children and adolescents. The PSC and the PSC-17 are efficient and quick screening tools, but the optimal cutoff value of the JPSC-17-Y for identifying children with psychosocial problems has not been determined. By establishing optimal cutoff values of the JPSC-17-Y in this study and implementing the JPSC-17-Y response form in school-provided tablet devices, prevention of and early intervention for psychosocial problems of schoolchildren could be achieved. Thus, this study aimed to determine the optimal cutoff value for identifying children with psychosocial problems for prevention and early intervention by comparing patients and normal controls.

## Methods

### Participants

The study involved 116 children, including 52 children with psycho-somatic-social problems, such as orthostatic dysregulation and irritable bowel syndrome, including missing school (23 boys and 29 girls aged from 12 to 15 years), and 64 age-matched normal control children (24 boys and 40 girls aged from 12 to 15 years) [Table 1]. The children with psycho-somatic-social problems were recruited from Kansai Medical University Medical Center, located in Osaka, Japan’s second-largest metropolitan area, whereas the control group was recruited from a private junior high school in the same city. The children with psycho-somatic-social problems were diagnosed with having any of the following symptoms or conditions: various somatic symptoms due to orthostatic intolerance, social maladaptation problems due to autism spectrum disorder and/or attention deficit/hyperactivity disorder, non-attendance at school, eating disorder, insomnia, severe obesity, and obsessive compulsive disorder. They were regarded as having psycho-somatic-social problems by specialists in developmental pediatrics when seen as outpatients. The exclusion criteria for both the children with psycho-somatic-social problems and the normal controls included: (i) holders of a mental disability or rehabilitation certificate, and (ii) absence of the ability to answer self-report questionnaires due to intellectual or cognitive problems. In contrast, no parental reports of psychosomatic or social problems had been received for the control group. The children with psycho-somatic-social problems completed the JPSC-17-Y either at treatment initiation or during the course of treatment, whereas the children in the control group completed the JPSC-17-Y in a classroom setting during class time. This study was conducted between July 2020 and September 2022, during which participants completed the JPSC-17-Y.

**Table 1:** Participants’ Characteristics

*N*	Patients	Controls
	52	64
Sex, M/F	23/29	24/40
Age, y	13.6 ± 0.9	13.4 ± 1.0

The patients and controls were junior high school students

### Measures

The PSC-17-Y is a validated measure, designed to evaluate psychosocial problems across three major psychopathological domains (internalizing, externalizing, and attention-deficit/hyperactivity disorder) in youths aged from 11 to 17 years [18]. This assessment tool is efficient, requiring only 5–10 min to complete. Participants are required to rate the frequency of each symptom on a 3-point Likert scale, which includes the options of 0 = never, 1 = sometimes, and 2 = often. The weighted scores are then summed to generate a total score that ranges from 0 to 34. In a study conducted by Murphy et al. [25] with a sample of 80,608 pediatric outpatients, reliability was high (internal consistency 0.89; test retest 0.85), and a confirmatory factor analysis provided support for the original 3-factor model. Similarly, the JPSC-17-Y showed high reliability (internal consistency 0.85; test–retest 0.86), and a confirmatory factor analysis supported the original 3-factor model [24]. Additional information regarding the PSC can be found at the following website.

[https://www.massgeneral.org/psychiatry/treatments-and-services/pediatric-symptom-checklist]

## Results

### Comparison of the JPSC-17-Y scores between the patient group and the control group

First, normality of each factor (attention factor, internalizing factor, externalizing factor, and the total score of the JPSC-17-Y) was assessed for each group. The results showed that the scores of the control group were not normally distributed for any of the factors. The Mann-Whitney U test was then used to examine the differences between the groups, and the results showed that the patient group had significantly higher scores than the control group for all factors and total scores [Table 2].

**Table 2:** Scores for each factor and the total score on the JPSC-17-Y

		NC	PT	U(114)=	*p*	Effect size(*r*)	[95% CI]		
		(*n* = 64)	(*n* = 52)						
attention	Mean	3	5.48	721.5	*p*<.001	-0.566	[-0.693,	-0.405	]
	SD	2.23	2.12						
exter- nalizing	Mean	2.75	3.77	1300.5	*p*<.05	-0.218	[-0.409,	-0.01	]
	SD	2.39	2.71						
inter- nalizing	Mean	3.14	5.29	950	*p*<.001	-0.429	[-0.586,	-0.242	]
	SD	2.87	2.64						
Sum	Mean	8.89	14.54	816	*p*<.001	-0.51	[-0.649,	-0.336	]
	SD	6.04	5.15						

The scores of the patient group (PT) were significantly higher than those of the control group (NC) for all factor and the total score

### Cutoff value

A logistic regression analysis was conducted to examine the predictive utility of the JPSC-17-Y for clinical diagnosis. The results showed that the JPSC-17-Y scores were significantly predictive of the clinical diagnosis (β = 0.172, SEB = 0.039, 95% confidence interval (CI) = [0.096, 0.249], odds ratio = 1.18). This finding was supported by the significant model fit, as indicated by the χ2 statistic (χ2 (144) = 25.35, *p*<.001) and McFadden’s R2 value (0.159). These results suggest that the score of the JPSC-17-Y is a meaningful predictor of the clinical diagnosis, and they demonstrate the importance of considering psychosomatic symptoms in diagnostic decision-making.

**Fig. 1:**
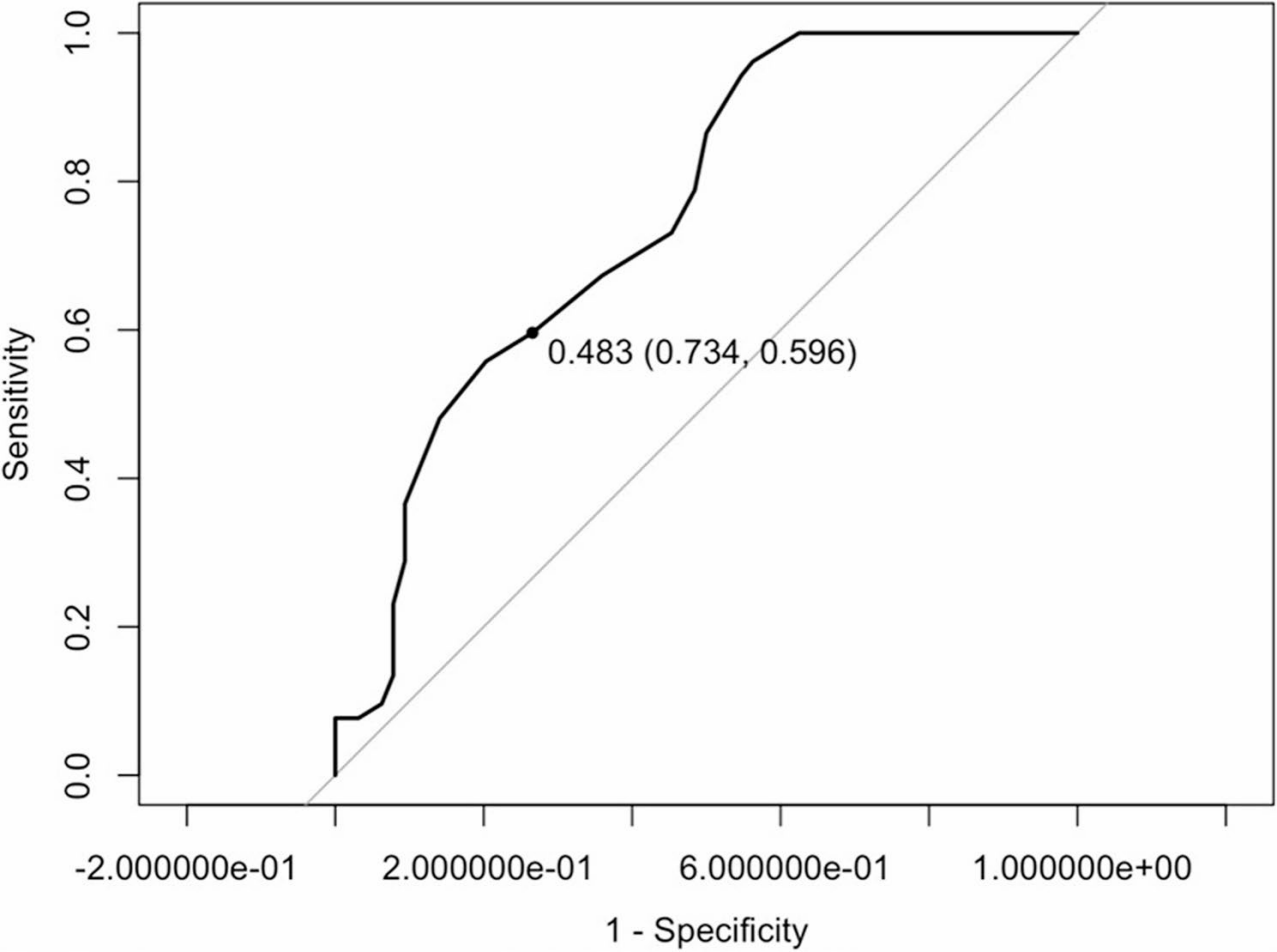
The Receiver-Operating Characteristic (ROC) curve for the JPSC-17-Y total score. For discrimination, sensitivity is 59.6%, specificity is 73.4%, positive predictive value is 64.6%, and negative predictive value is 69.1%

To determine the cutoff value for differentiating between the control group and the patient group using the total score of the JPSC-17-Y, the Area Under the Curve (AUC) of the Receiver Operating Characteristic (ROC) curve was calculated. The AUC was 0.755, indicating a moderate level of predictive performance for the JPSC-17-Y. The closest top-left index had the JPSC-17-Y total score range between 12 and 13. The results are presented in Fig. 1, based on the aforementioned cutoff values. In the patient group, there were 52 participants, of whom 31 tested positive and 21 tested negative. In contrast, in the control group, 17 participants tested positive and 47 tested negative. For discrimination, sensitivity was 59.6%, specificity was 73.4%, the positive predictive value was 64.6%, and the negative predictive value was 69.1%. It is important to note that the accuracy of the JPSC-17-Y may not always be high due to the nature of psychosomatic disorders. Some schoolchildren in the control group may have problems, whereas some patients in the patient group may show improved symptoms. Despite these limitations, the JPSC-17-Y remains an informative tool that can provide important diagnostic information.

## Discussion

In the present study, scores of the JPSC-17-Y were compared between pediatric patients and normal controls to determine the optimal cutoff value for identifying children with psychosocial problems for prevention and early identification.

The score of each factor and the total score of the JPSC-17-Y were significantly higher than in the patient group than in normal controls. In addition, the 12/13 cutoff value of the total score showed high sensitivity and specificity for identifying children with psychosocial problems.

### Difference of the cutoff value between the Japanese version and the original version

In the present study, the optimal cutoff value of the JPSC-17-Y for identifying children with psychosocial problems for prevention and early treatment was found to be 12/13. In contrast, the cutoff value is 15/16 for the original PSC-17-Y. That is, the JPSC-17-Y has a relatively lower cutoff value. What could be the potential factors contributing to this difference in cutoff values between the original and Japanese versions?

Even in a 35-item original version of the PSC, the cutoff value for Japanese children was significantly lower than that of the original version. Ishizaki et al. [26] stated the reasons for this as follows: (i) the quality of psychosocial problems is different in Japan and the United States, with the severity being lower in Japan, and (ii) Japanese people tend to choose the middle answer for questions with three levels (central tendency), and they point out that there are few answers for “often”. The same pattern was observed in the participants of the present study.

### Limitations

Several limitations of this study need to be considered. The scores in the normal control group may have been lower due to the difference in implementation. The normal control group answered the JPSC-17-Y in the presence of their peers at school, whereas the patient group completed it alone within the hospital setting. It is plausible that some schoolchildren in the normal control group may have scored below their actual potential due to answering the JPSC-17-Y in a group setting. Consequently, this could have potentially contributed to a decreased total score on the JPSC-17-Y and subsequently the lower cutoff value than that of the original PSC-17-Y.

Other limitations include the small sample size and single-center sampling. In the future, it will be necessary to collect and analyze larger samples.

### Future research

Whereas the current study focused on children aged 12 to 15 years attending junior high school, further research is needed to examine the validity of the JPSC-17-Y and establish a cutoff value for schoolchildren of upper elementary school. Early detection of psychosocial problems and timely intervention can be facilitated by such an approach. In addition, if differences in high factor scores are observed between schoolchildren attending elementary school and junior high school, tailored interventions may be necessary.

In addition, children with either a low intellectual quotient or those with high-functioning autism spectrum disorder demonstrated significant difficulty in providing accurate responses to the JPSC-17-Y assessment. Children with intellectual and developmental disabilities are more susceptible to experiencing challenges and obstacles. Thus, future research should investigate the possibility of accurate implementation of the assessment of handicapped children by having testers read questions to the participants or by providing additional clarification regarding the intent of the questions.

Finally, implementation of online screening using the JPSC-17-Y is a goal of our future research. Murphy et al. [27–28] and Arauz-Boudreau et al. [29] reported the usefulness of an internet-based approach using PSC-Y and PSC-17 Form. We implemented the JPSC-17-Y response form on school-provided tablet devices in several junior high schools within a single prefecture as part of a pilot initiative. We are also currently investigating the usefulness of online screening combined with AI chat and the JPSC-17-Y [30]. Several children who exhibited high total scores of the JPSC-17-Y response form were subsequently referred for school counseling services. As this initiative is scaled up, it may hold increasing potential to reduce the incidence of non-attendance at school and suicide. This is an attempt to evaluate and improve the mental health of children who are self-isolating at home due to non-attendance at school, and it is expected to be used as a psychosocial screening tool for children who are not attending school to early identification and intervention.

## Conclusions

The score for each factor and the total score of the JPSC-17-Y were significantly higher in the patient group than in normal controls. In addition, the 12/13 cutoff value of the total score showed high sensitivity and specificity. The JPSC-17-Y response form was implemented on school-provided tablet devices. As a result, several children who exhibited high total scores on the JPSC-17-Y response form were subsequently referred for school counseling services. These findings support that the use of the JPSC-17-Y for the prevention of and early intervention for psychosocial problems of schoolchildren.

## Supplementary Information

Supplementary Material 1.

## Data Availability

All data generated or analyzed during this study are included in this published article and its supplementary information files.

## Abbreviations

PSC: The Pediatric Symptom Checklist
PSC-17-Y: 17-item version of the PSC-Youth Self-Report
JPSC-17-Y: the Japanese version of the PSC-17-Y
AUC: Area Under the Curve
ROC: Receiver Operating Characteristic
